# Transmission of malaria in relation to distribution and coverage of long-lasting insecticidal nets in central Côte d’Ivoire

**DOI:** 10.1186/1475-2875-13-109

**Published:** 2014-03-19

**Authors:** Allassane F Ouattara, Mamadou Dagnogo, Edi AV Constant, Moussa Koné, Giovanna Raso, Marcel Tanner, Piero L Olliaro, Jürg Utzinger, Benjamin G Koudou

**Affiliations:** 1 Centre Suisse de Recherches Scientifiques en Côte d’Ivoire, 01 BP 1303, Abidjan 01, Côte d’Ivoire; 2 Université Nangui Abrogoua, 02 BP 801, Abidjan 02, Côte d’Ivoire; 3 Department of Epidemiology and Public Health, Swiss Tropical and Public Health Institute, P.O. Box, CH–4002, Basel, Switzerland; 4 University of Basel, P.O. Box, CH–4003, Basel, Switzerland; 5 Vector Biology Department, Liverpool School of Tropical Medicine, Liverpool L3 5QA, UK; 6 Centre d’Entomologie Médicale et Vétérinaire, Université de Bouaké, Bouaké, Côte d’Ivoire; 7 UNICEF/UNDP/World Bank/WHO Special Programme for Research and Training in Tropical Diseases (TDR), 20 avenue Appia, CH–1211 Geneva 27, Switzerland; 8 Centre for Neglected Tropical Diseases, Liverpool School of Tropical Medicine, Liverpool L3 5QA, UK

**Keywords:** Malaria, Transmission, Entomology, Long-lasting insecticidal net, Côte d’Ivoire

## Abstract

**Background:**

The use of long-lasting insecticidal nets (LLINs) is an effective malaria control strategy. However, there are challenges to achieve high coverage, such as distribution sustainability, and coverage keep-up. This study assessed the effect of LLINs coverage and contextual factors on entomological indicators of malaria in rural Côte d’Ivoire.

****Method**s:**

The study was carried out between July 2009 and May 2012 in three villages (Bozi, N’Dakonankro and Yoho) of central Côte d’Ivoire. In Bozi and Yoho, LLINs were distributed free of charge by the national malaria control programme in 2008. In Bozi, an additional distribution was carried out in May 2011. No specific interventions were done in N’Dakonankro. Entomological surveys were conducted in July 2009 and July 2010 (baseline), and in August and November 2011 and in February 2012. Frequency of circumsporozoite protein was determined using an enzyme-linked immunosorbent assay. Regression models were employed to assess the impact of LLINs and changing patterns of irrigated rice farming on entomological parameters, and to determine associations with LLINs coverage and other contextual factors.

**Results:**

In Bozi, high proportion of LLIN usage was observed (95-100%). After six months, 95% of LLINs were washed at least once and 79% were washed up to three times within one year. *Anopheles gambiae* was the predominant malaria vector (66.6% of all mosquitoes caught). From 2009 to 2012, in N’Dakonankro, the mean annual entomological inoculation rate (EIR) increased significantly from 116.8 infectious bites/human/year (ib/h/y) to 408.8 ib/h/y, while in the intervention villages, the EIR decreased significantly from 514.6 ib/h/y to 62.0 ib/h/y (Bozi) and from 83.9 ib/h/y to 25.5 ib/h/y (Yoho). The risk of an infectious bite over the three-year period was significantly lower in the intervention villages compared to the control village (p <0.001).

**Conclusion:**

High coverage and sensitization of households to use LLINs through regular visits (particularly in Bozi) and abandoning irrigated rice farming (in Yoho) resulted in highly significant reductions of EIR. The national malaria control programme should consider household sensitization and education campaigns and other contextual factors to maximize the benefit of LLINs.

## Background

In terms of at-risk population, mortality and global burden, malaria remains the most important vector-borne disease worldwide [1,2]. Over the past 15 years, considerable progress has been made in the control of malaria, and elimination has been declared as the ultimate goal [3]. However, there are huge challenges ahead, such as gaining and sustaining high coverage rates with tools of proven efficacy for vector control, including long-lasting insecticidal nets (LLINs) [4] and indoor residual spraying (IRS) [5]. In high transmission areas, the use of LLINs is an effective strategy to reduce human-vector contacts [6-8]. Hence, LLINs are considered an essential tool to prevent malaria [9,10], that however requires high coverage and proper use [11,12]. Costs are an important barrier to reach high coverage with LLINs, particularly in rural areas of sub-Saharan Africa [13,14]. To overcome this obstacle, the free distribution of LLINs has been suggested as a means to obtain high ownership rates [15,16]. Nonetheless, it has been observed that ownership does not necessarily translate into effective usage [15]. Moreover, nets might be used only during certain times of the year, for example during the rainy season for protection against the nuisance of *Culex* mosquitoes [17]. In many countries, national malaria control programmes continue to distribute LLINs free of charge, perhaps without sufficient attention given to issues of distribution sustainability and coverage keep-up, which in turn are necessary to improve the effectiveness of LLINs. Few studies have investigated how a high LLIN coverage could be maintained [18,19]. Such research is necessary to achieve high coverage and a high proportion of usage in settings where intervention success is strongly correlated with local concepts and beliefs regarding how malaria is transmitted.

In Côte d’Ivoire, malaria transmission is perennial [1]. Since 2010, the national malaria control programme, with the support of the ‘Global Fund to Fight AIDS, Tuberculosis and Malaria’ (Global Fund), started scaling up mass distribution of LLINs. Previous research in several districts of the country emphasised that malaria transmission is governed by strongly rooted socio-cultural beliefs and local concepts [20-22], and these should be taken into consideration by the programme.

The current study aimed to assess the effect of coverage and use of LLINs on *Plasmodium* transmission in three villages of central Côte d’Ivoire. Contextual factors, such as changes in agricultural practices, were also investigated. Entomological data were collected prospectively in a series of cross-sectional surveys.

## Methods

### Ethics statement

The study protocol was reviewed by the institutional review board of the Centre Suisse de Recherches Scientifiques en Côte d’Ivoire (CSRS; Abidjan, Côte d’Ivoire) and approval was given by the national ethics committee of Côte d’Ivoire (reference no. 02-2011/MSLS/CNER-P). Mosquito collectors were immunized against yellow fever and received recommended first-line anti-malarial chemoprophylaxis. Oral informed consent was obtained from heads of households with regard to their willingness to participate in the study. Oral rather than written informed consent was sought due to high illiteracy among the adult population in this part of Côte d’Ivoire. The purpose, procedures, potential risks and benefits of the study were explained in the local language using lay terms. Participation was voluntary, and hence, people could withdraw anytime from the study without further obligation.

### Study area

**Figure 1:**
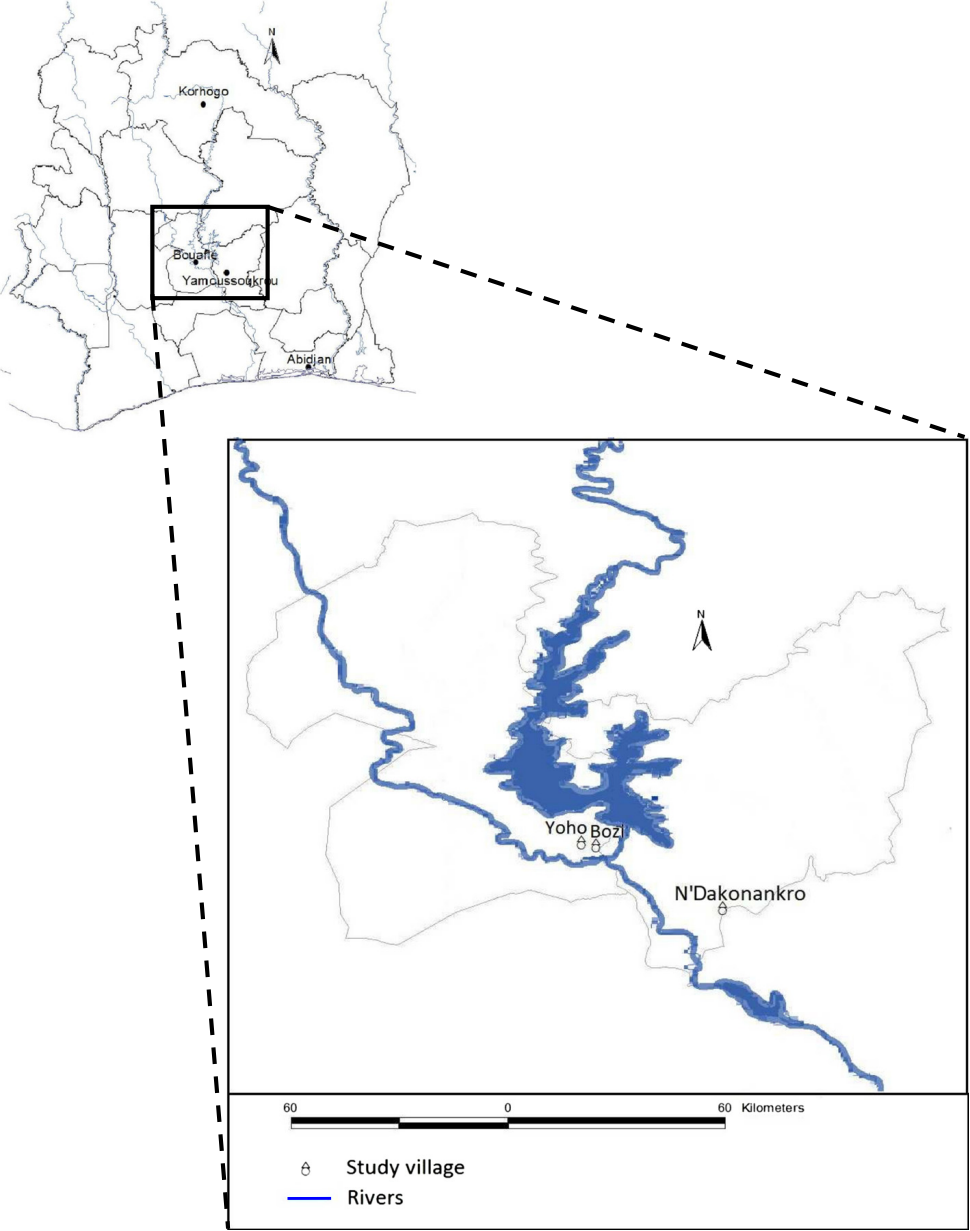
Study site location.

The study was carried out between July 2009 and May 2012 in three villages of central Côte d’Ivoire. N’Dakonankro (geographical coordinates 6°45.560’ N latitude and 5°13.195’ W longitude) is located near Yamoussoukro, the political capital of Côte d’Ivoire. The monthly temperature ranges between 27°C and 29°C with a mean humidity of 70-80% in the rainy season. The mean annual precipitation during 2009–2011 was 1,181 mm with peaks observed between mid-March and mid-July and September/October (SODEXAM, 2012). Bozi (6°55.151’ N, 5°32.080’ W) and Yoho (6°55.364’ N, 5°34.569’ W) are two villages separated by a distance of only 5 km and are located in the department of Bouaflé (Figure 1). The average annual temperature is 26°C, the mean relative humidity is 75%, and the mean annual precipitation during 2009–2011 was 1,236 mm. Both villages use the same health facility. At the beginning of this study, irrigated rice farming was practiced in all three villages in close proximity to human settlements.

### Study design and timing

**Figure 2:**
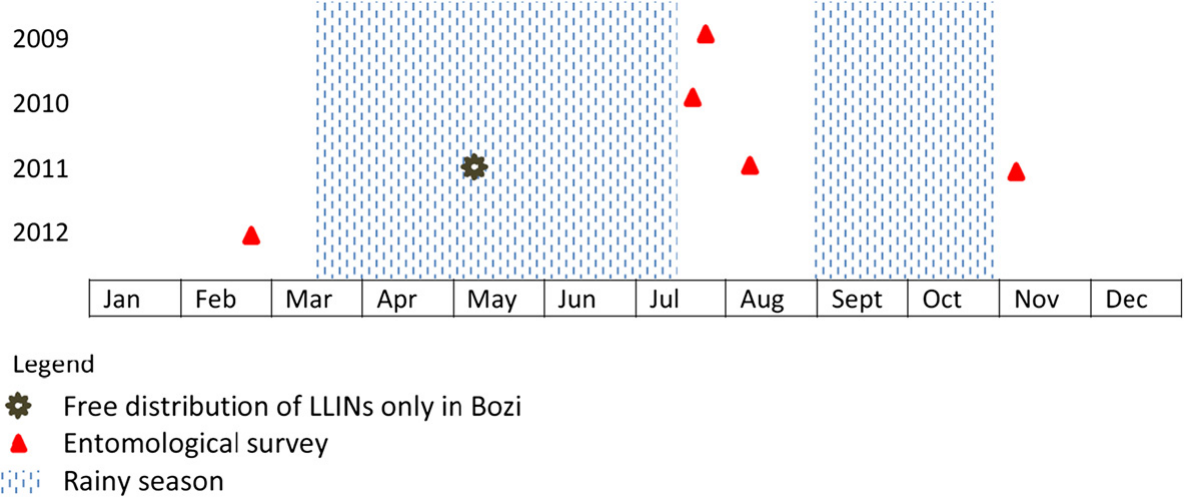
Study design and timing of entomological surveys and free distribution of LLINs from 2009 to 2012 in three villages of central Côte d’Ivoire.

Figure 2 shows the design of the study, emphasising the timing of the entomological surveys and the distribution of LLINs. In brief, human-bait night catches for adult mosquito collection were done in July 2009 and July 2010 before LLIN distribution, and again after LLIN distribution in August and November 2011 and in February 2012. Additionally, larval collections were done at each occasion. Once every month, the physical conditions and use of LLINs were assessed.

### Free LLIN distribution and household visits

In Bozi and Yoho, LLINs were distributed free of charge by the national malaria control programme. However, in early 2009, after the mass distribution of LLIN, a survey revealed low coverage rates – 35.2% in Bozi and 10.2% in Yoho [21]. In N’Dakonankro, LLINs had not been distributed during the study period, and hence this village served as a control. The reason why N’Dakonankro did not benefit from the mass distribution of LLINs was that the district of Yamoussoukro intended to run an IRS campaign, which, however, never materialized. A survey found bed net coverage as low as 7.1% in N’Dakonankro.

In May 2011, with the help of community health workers (CHWs), an additional 150 LLINs were distributed, free of charge, to households in Bozi. The aim was to reach an overall coverage of 80%. LLINs were primarily distributed to households where at least one child below the age of 15 years or a pregnant woman resided. Heads of households were sensitized about net usage and washing procedures. Three months after this second LLIN distribution in Bozi, CHWs commenced with a longitudinal monitoring, investigating the physical conditions and use of LLINs by household visits once every month.

### Larval collections

*Anopheles* and *Culex* larvae were sampled in potential mosquito breeding sites previously identified in the study villages, adhering to standard protocols [23]. In brief, a 350-ml dipper was used to draw 10 samples (“dips”) from each site. All breeding sites within a 3-km radius around each village were investigated. Collected mosquito larvae were stored in 5-l containers. Larvae were identified and grouped at genus level. Potential larval habitats were characterised, using a rapid appraisal tool previously developed and validated in Côte d’Ivoire [23].

### Adult mosquito collection

During each survey, mosquito collection was carried out inside and outside of randomly selected sentinel houses (six houses per village, three for indoor and three for outdoor collections), during two consecutive nights between 18:00 and 06:00 hours, using human-bait catches. After collection, mosquitoes were counted and morphologically identified using Mattingly’s taxonomic key [24]. Mosquitoes were stored individually in tubes with silica gel and kept at −20°C pending further laboratory processing.

### Pyrethrum spray collection

Windows of sleeping rooms from selected houses were closed in the morning and oral consent of heads of households was obtained. Eight houses per village during the baseline (2009–2010) and 15 houses per village during the intervention (2011–2012) were selected early in the morning (06:00 hours). A white bed sheet was laid on the floor and a pyrethrum insecticide was sprayed in the bedrooms. After 10 min, the sheet was removed and the dead mosquitoes were classified according to their physiological status, placed in tubes and transferred to a nearby laboratory.

### Determining *Plasmodium falciparum* infection in female mosquitoes

The head and thorax of mosquitoes belonging to the genus *Anopheles* were analysed for circumsporozoite protein, using an enzyme-linked immunosorbent assay (ELISA) [25]. The optical density (OD) is proportional to the amount of circumsporozoite protein in the ELISA plate wells from a single mosquito and the threshold of positivity was determined at the mean OD of five negative controls.

### Statistical analysis

Statistical analyses were performed with STATA version 10.4 (Stata Corporation; College Station, TX, USA). The density of larvae was calculated and expressed as the number of larvae per liter in a given breeding site and averaged for the unit of a village. The biting rate was the average number of adult female mosquitoes collected through human-bait catches. Sporozoite index, expressed as a percentage, was the number of circumsporozoite protein-positives out of the number of tested mosquitoes. Entomological inoculation rate (EIR) was the biting rate multiplied by the sporozoite index. The density of mosquitoes resting indoor was the average number of *Anopheles* collected inside a house during the baseline (2009–2010) and the intervention (2011–2012), as assessed by pyrethroid spray catches.

One-way Anova was used to express differences in the number of larvae collected in the study villages and Student’s *t*-test was used to compare larval densities. Random effect negative binomial regression models were employed to compare the biting rate of mosquitoes between villages. Village difference in mosquitoes’ sporozoite index was analysed using random effect logistic regression models. Poisson regression models were used for EIR analyses. Indoor resting densities of mosquitoes were compared between villages using a Kruskal-Wallis test (H), with a p-value <0.05 considered for statistical significance. Chi-square (χ^2^) or Fisher’s exact test, as appropriate, were used to compare groups. Model coefficients were expressed as incidence risk ratio (IRR) or odds ratio (OR) with corresponding 95% confidence intervals (CIs). Statistical difference was reached when 1 was not included in the 95% CIs.

## Results

### Usage of LLINs and conditions after 12 months

In Bozi, the proportion of LLIN used ranged between 95% and 100% between May 2011 and May 2012. At the end of 2011, assessment of the physical condition of LLINs revealed that 2-4% of LLINs were torn, 1-2% had holes and 95% of LLINs had been washed at least once. By May 2012, 79% of the nets had been washed at least three times (Table 1).

**Table 1:** Usage and care of LLINs distributed free of charge in households from Bozi over a one-year period

	**Installation no. (%)**			**Condition no. (%)**		**No. of times washed (%)**			
	**Installed**	**Not installed**	**Total**	**Torn**	**Holes**	**0**	**1**	**2**	**3**
**2011**									
August	143 (95)	7 (5)	150	3 (2)	2 (1)	89 (59)	49 (33)	5 (3)	0 (0)
September	146 (97)	4 (3)	150	6 (4)	3 (2)	2 (1)	139 (93)	5 (3)	0 (0)
October	147 (98)	3 (2)	150	3 (2)	0 (0)	0 (0)	142 (95)	5 (3)	0 (0)
November	144 (96)	6 (4)	150	3 (2)	3 (2)	0 (0)	139 (93)	5 (3)	0 (0)
December	144 (96)	6 (4)	150	3 (2)	3 (2)	0 (0)	2 (1)	142 (95)	0 (0)
**2012**									
January	150 (100)	0 (0)	150	3 (2)	3 (2)	0 (0)	2 (1)	148 (99)	0 (0)
February	147 (98)	3 (2)	150	1 (1)	1 (1)	0 (0)	1 (1)	146 (97)	0 (0)
March	147 (98)	3 (2)	150	2 (1)	2 (1)	0 (0)	0 (0)	125 (83)	22 (15)
April	147 (98)	3 (2)	150	3 (2)	2 (1)	0 (0)	0 (0)	51 (34)	96 (64)
May	147 (98)	3 (2)	150	3 (2)	3 (2)	0 (0)	0 (0)	28 (19)	119 (79)

### Characteristics of mosquito larval breeding sites

**Figure 3:**
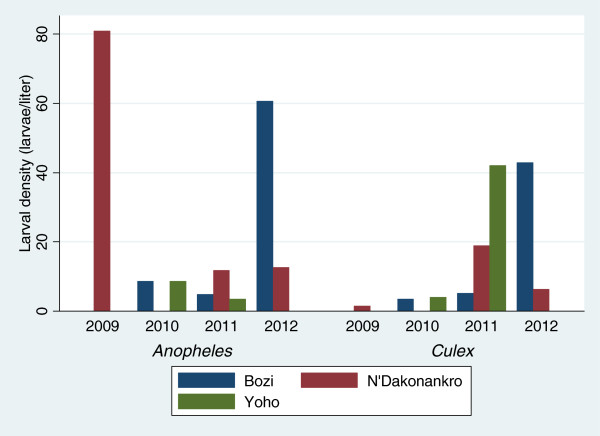
Larval density stratified by mosquito genus and year of survey in three villages of central Côte d’Ivoire.

A wide variety of larval breeding sites were identified, including irrigated rice fields, irrigation channels or wells, lowlands and animal foot prints, especially from oxen. In Yoho, the frequency of irrigated rice farming was reduced from 2009 to 2010 and was completely interrupted in 2011 and 2012. In 2010, *Anopheles* and *Culex* larvae were found in all potential breeding sites. During the fourth quarter of 2011, no larvae were found in any of the breeding sites (Figure 3). In Bozi, *Anopheles* and *Culex* larvae were collected in 75% of the breeding sites in 2010. In 2012, only one breeding site (lowland) contained both larvae and pupae. In N’Dakonankro, rice production was maintained throughout the study period and the main source of larval production was an irrigated rice field located in close proximity to households (≤120 m).

### Species composition and abundance of Culicidae fauna

Total sampling attempts were 24 human bait-night catches during the baseline (2009–2010) and 36 human bait-night catches during the intervention (2011–2012). The number of mosquitoes caught during the baseline and the intervention periods in N’Dakonankro, Bozi and Yoho were 3,807, 1,433 and 774, respectively.

In N’Dakonankro, Yoho and Bozi, *Anopheles* represented 72.1% (n = 2,743), 89.3% (n = 691) and 93.0% (n = 1,333) of all mosquitoes caught, respectively. *An. gambiae* was the predominant species (66.6% overall). It accounted for 57.0% in N’Dakonankro, 83.3% in Yoho and 83.4% in Bozi. The abundance of other mosquito species (*Mansonia* spp. and *Culex* spp.) ranged between 10% and 30% (Additional files 1, 2 and 3).

### Biting rate

During the baseline survey, *An. gambiae* biting rates of 16.2, 23.5 and 35.3 bites/human/night (b/h/n) were observed in N’Dakonankro, Yoho and Bozi, respectively (Table 2). During the intervention period, the mean biting rates recorded were 49.3, 2.2 and 9.6 b/h/n in these three villages, respectively, corresponding to a three-fold increase, and to a 91% and 73% decrease compared to the baseline values, respectively (Table 2). Table 3 shows the results of the negative binomial regression comparing *An. gambiae* biting rates between villages (N’Dakonankro as the control) with capture location considered as random effect. During the baseline survey, the risk of being bitten by *An. gambiae* was 1.3 times higher in Bozi and 1.2 higher in Yoho compared to N’Dakonankro. These differences lacked statistical significance (p >0.05). After the intervention period, the risk of being bitten by *An. gambiae* in Bozi or Yoho was 0.15 (95% CI 0.09-0.24) and 0.05 (95% CI 0.03-0.10) compared to that in N’Dakonankro (both p <0.001).

**Table 2:** **Entomological parameters of *An. gambiae *** **stratified by study period in three villages of central Côte d’Ivoire**

	**Captured number**	**Biting rate (b/h/n)**	**Tested (infected)**	**Sporozoite index (%)**	**EIR (ib/h/n)**	**Annual EIR (ib/h/year)**
2009-2010 period						
N’Dakonankro	389	16.2 (9.8-22.6)	100 (2)	2.0 (0–4.8)	0.32 (0–1.08)	116.8 (0–394.2)
Indoor	259	21.6 (9.7-33.4)	50 (2)	4.0 (0–9.6)	0.86 (0–3.22)	313.9 (0–1,175.3)
Outdoor	130	10.8 (5.5-16.1)	50 (0)	0 (0–7.0)*	0 (0–1.13)*	0 (0–412.5)*
Yoho	565	23.5 (14.3-32.8)	100 (1)	1.0 (0–3.0)	0.23 (0–0.98)	83.9 (0–357.7)
Indoor	320	26.7 (10.5-42.8)	50 (0)	0 (0–7.0)*	0 (0–3.00)*	0 (0–1,095.0)*
Outdoor	245	20.4 (8.7-32.1)	50 (1)	2.0 (0–6.02)	0.41 (0–1.93)	149.6 (0–704.4)
Bozi	848	35.3 (21.9-48.7)	100 (4)	4.0 (0.1-7.9)	1.41 (0.02-3.85)	514.6 (7.3-1,405.2)
Indoor	421	35.1 (13.7-56.5)	49 (1)	2.0 (0–6.1)	0.72 (0–3.47)	262.8 (0–1,266.5)
Outdoor	427	35.6 (15.8-55.4)	51 (3)	5.9 (0–12.6)	2.09 (0–6.96)	762.8 (0–2,540.4)
2011-2012 period						
N’Dakonankro	1,776	49.3 (38.0-60.7)	1,101(25)	2.3 (1.4-3.2)	1.12 (0.53-1.91)	408.8 (193.4-697.1)
Indoor	712	39.5 (27.0-52.1)	497 (18)	3.6 (2.0-5.3)	1.43 (0.54-2.74)	521.9 (197.1-1,000.1)
Outdoor	1,064	59.1 (39.9-78.3)	604 (7)	1.2 (0.3-2.0)	0.69 (0.12-1.57)	251.8 (43.8-573.0)
Yoho	80	2.2 (0.1-4.4)	59 (2)	3.4 (0–8.1)	0.07 (0–0.35)	25.5 (0–127.7)
Indoor	18	1.0 (0–2.3)	14 (0)	0 (0–26.0)*	0 (0–0.59)*	0 (0–215.3)*
Outdoor	62	3.4 (0–7.7)	45 (2)	4.4 (0–10.7)	0.15 (0–0.82)	54.7 (0–299.3)
Bozi	347	9.6 (3.4-15.9)	230 (4)	1.7 (0–3.4)	0.17 (0–0.55)	62.0 (0–200.7)
Indoor	120	6.7 (1.2-12.1)	83 (2)	2.4 (0–5.8)	0.16 (0–0.70)	58.4 (0–255.5)
Outdoor	227	12.6 (0.8-24.4)	147 (2)	1.4 (0–3.2)	0.18 (0–0.79)	65.7 (0–288.3)

( ): 95% confidence interval.

( )*: 97.5% confidence interval.

b/h/n: bites/human/night.

ib/h/n: infectious bites/human/night.

**Table 3:** **Results of regression models on entomological parameters of *An. gambiae *** **(random effect “indoor or outdoor”)**

	**Biting rate**	**Sporozoite index**	**EIR**^**#**^
**Variables**	**IRR**^**§ **^(95% CI^*^)	**Odds ratio (95% CI)**	**IRR (95% CI)**
2009-2010 period			
N’Dakonankro	1.00	1.00	1.00
Yoho	1.19 (0.75-1.90)	0.49 (0.04-5.54)	0.47 (0.55-4.04)
Bozi	1.31 (0.82-2.08)	2.04 (0.36-11.40)	3.25 (0.60-17.63)
2011-2012 period			
N’Dakonankro	1.00	1.00	1.00
Yoho	0.05 (0.03-0.10)^†^	1.75 (0.40-7.69)	0.07 (0.0-0.36)^†^
Bozi	0.15 (0.09-0.24)^†^	0.81 (0.28-2.35)	0.16 (0.09-0.27)^†^

^§^IRR: incidence risk ratio.

^*^CI: 95% confidence interval.

^#^EIR: entomological inoculation rate.

^†^Significant.

### Indoor resting mosquito density (endophily rate)

During the baseline period, the density of *An. gambiae* resting indoor was, on average, 2.0 females per house (f/h) in N’Dakonankro, 4.1 f/h in Yoho and 7.5 f/h in Bozi. During the intervention period, the endophily rates in Bozi and Yoho were estimated at 0.4 f/h (a 95% reduction rate) and 2.4 f/h (a 41% reduction rate) and were in both cases significantly lower (p <0.05) than the one recorded in N’Dakonankro (7.1 f/h).

### Infection rate (sporozoite index)

*Anopheles gambiae* infection rates were 2.0%, 1.0% and 4.0% in N’Dakonankro, Yoho and Bozi, respectively during the baseline period, and remained stable in N’Dakonankro (2.3%), increased in Yoho (3.4%) and decreased only in Bozi (1.7%, corresponding to a 57% drop) during the intervention period (Table 2). Though no statistically significant difference between sporozoite rates was recorded on either occasion by negative binomial regression, a reduction of infection rate was recorded only in Bozi (Table 3). No *Anopheles funestus* female were found infected in any of the study villages.

### Entomological inoculation rate (EIR)

During the baseline survey, the mean annual EIR was estimated at 116.8 infectious bites/human/year (ib/h/y) (313.9 ib/h/y indoor *vs.* 0 ib/h/y outdoor), 83.9 ib/h/y (0 ib/h/y indoor *vs.* 149.6 ib/h/y outdoor), 514.6 ib/h/y (262.8 ib/h/y indoor *vs.* 762.8 ib/h/y outdoor) in N’Dakonankro, Yoho and Bozi, respectively. During the intervention period, the respective EIRs were 408.8 ib/h/y (521.9 ib/h/y indoor *vs.* 251.8 ib/h/y outdoor), 25.5 ib/h/y (0 ib/h/y indoor *vs.* 54.7 ib/h/y outdoor) and 62.0 ib/h/y (58.4 ib/h/y indoor *vs.* 65.7 ib/h/y outdoor) (Table 2), corresponding to a 3.5 fold increase in N’Dakonankro, a reduction by 70% in Yoho and a reduction of 88% in Bozi. Before the intervention, the EIR was three times higher outdoors than indoors.

IRRs for infectious bites between villages were not significantly different at baseline (Table 3). However, from 2011 to 2012, the risk of receiving an infectious bite was highly significantly lower in Bozi (IRR = 0.16, 95% CI, 0.09-0.27) and Yoho (IRR = 0.07, 95% CI, 0.01-0.36) compared to N’Dakonankro.

## Discussion

This study investigated entomological indicators of malaria in three villages of central Côte d’Ivoire (five surveys carried out over a three-year period), and showed the worst baseline entomological indexes in Bozi, a role played by household’s sensitization to increase the use of LLINs and a broader community effect through mass distribution of LLIN. Indeed, in N’Dakonankro biting rates and EIR increased more than three times (from 16.2 to 49.3 b/h/n and from 116.8 to 408.8 ib/h/y, respectively); infections occurred largely indoors. Here, no LLIN distribution or other malaria control interventions were deployed, and no change in environment or agricultural habit occurred. The absence of malaria control interventions resulted in a further deterioration of an already critical malaria situation.

In Bozi, only a small proportion of LLINs were found holed (1-2%) or torn (2-4%) after one year of utilization. For comparison, in western Kenya, as much as 40% of nets were reported to be in poor physical conditions a year after distribution [26]. There is evidence that conditions of the nets are important for personal protection [27], that holed LLINs (or insecticide-treated nets (ITNs)) are less effective than untreated nets without holes [28], and that bed net use decreases with increasing physical damage of nets [29]. The use of LLINs in Bozi reached 100% due to several factors like household training to maintain good conditions of nets [30,31] and newness of nets [32].

Breeding sites rearing *Anopheles* and *Culex* larvae were frequent throughout the study sites. Irrigated rice fields were the main source of larval production located in close proximity to households. The same observation has been reported in Mali where growing stages of irrigated rice were correlated to *An. gambiae* larvae abundance, but larvae were non-existent in abandoned rice paddies [33]. Water pools and puddles were productive habitat types [34] similar to what was found in the study sites investigated here (e.g. animal foot prints and lowland).

*Anopheles* was the most prevalent genus (72.1-93.0%), 66.6% of which was *An. gambiae. Mansonia* spp. and *Culex* spp. accounted for the remaining 10-30% of adult mosquitoes collected. Similar findings have been reported in western Côte d’Ivoire where 51.4% of mosquitoes collected were *Anopheles* with 65.2% of *An. gambiae*[35], and in rural areas of Burkina Faso (92.7% were *Anopheles* with 84% of *An. gambiae*[36]). Importantly, *An. gambiae* remains the predominant species after net distribution [37]. Inversely, in southern coastal Kenya, Culicines predominate (95.1%) [38].

In Yoho, throughout the study period, the reductions in biting rate and EIR were approximately 90% and 70%, respectively and transmission was exclusively outdoors. Yoho received a regular supply of LLINs (but unlike Bozi, no extra mass intervention); in the second part of the period under observation, irrigated rice farming was interrupted in the immediate vicinity of the village, thus removing potential breeding sites.

In the first part of the study, Bozi had the worst entomological indexes: the number of captures and biting rates were nearly twice and one and a half as many as in N’Dakonankro and Yoho, respectively, and the EIR was more than four and six times, respectively. Bozi was subjected to an additional LLIN distribution campaign, after which the number of captures more than halved, biting rates dropped by 73% and EIR declined by 88%. The largest drop in EIR was for outdoor biting; the risk of infection was three times higher outdoors than indoors before, and became almost the same after the intervention. Increased outdoor feeding after the distribution of ITNs has been reported in Tanzania and Equatorial Guinea [39,40]. The overall reduction in EIR in Bozi was significantly greater than in Yoho and N’Dakonankro. No apparent environmental changes occurred here.

Household sensitization to increase the use of LLINs, and longitudinal monitoring over the course of the post-intervention follow-up in Bozi are likely to have played a role, explaining the considerable reduction in EIR compared to Yoho. However, these measures do not appear to afford long-lasting effects: a subsequent census revealed low coverage rates in both villages (35.2% in Bozi and only 10.2% in Yoho). Conversely, in Yoho, where coverage rate of LLINs remained low despite a free distribution, abandoning irrigated rice farming might explain the significant reduction in EIR, as observed elsewhere in Côte d’Ivoire [41,42]. Moreover the increased motivation for sleeping under LLINs in Bozi could be related to the extra distribution coinciding with the main rainy season (May-June 2011), when biting rates are particularly high. Importantly, previous studies carried out in three large towns of West Africa (Abidjan, Cotonou and Ouagadougou) during periods of high biting rates demonstrated that sleeping under ITNs the night before the survey was correlated with protection against *Plasmodium* infection [43-45]. Additionally, after LLIN distribution, CHWs sensitized households and monitored LLIN usage within households. An idea might be that the motivation for using LLINs was high just after household-to-household sensitization campaigns. This hypothesis is confirmed by findings from a recent study carried out in Burkina Faso, which showed that the motivation for the use of LLINs decreased 10 months after their distribution and when mosquito biting rates were low [11].

Preceding research carried out in a village of northern Côte d’Ivoire found that the rate of malaria attacks was twice as high among non-users of nets. Meanwhile, in previous studies conducted in Côte d’Ivoire, the differences in protective efficacies against uncomplicated malaria between ITN and non-ITN-users were low [46].

Interestingly, in a recent study, the presence of LLIN and history of sleeping under LLIN the night or week preceding the survey, as well as appropriate use of LLINs, showed no significant association with clinical malaria [47]. Two points are offered that might explain this surprising observation. First, these might be setting-specific difference in considering appropriate use of LLINs. Second, the presence of LLIN does not guarantee appropriate utilisation, and hence long-term protection from malaria [47]. Thus, the present study underscores that in malaria-endemic countries, national control programmes should accompany mass distribution of LLIN with setting-specific sensitization, monitoring of LLIN usage, and effective mechanisms to encourage people to replace damaged nets in a timely manner.

The current study also highlighted a broader community effect through mass distribution of LLIN and changing contextual factors in highly malaria-endemic settings. In fact, in Yoho where mass distribution of LLIN was carried out by the national malaria control programme, but reached only low coverage in addition to changing patterns of irrigated rice farming, the mean number of blood-fed *An. gambiae* resting indoor was significantly lower compared to the one recorded in the non-intervention village. The presence of pyrethroid-based LLINs in Bozi and Yoho that have an excito-repellent effect adds to a chemical barrier, reducing vectors populations [48,49]. The same observation was made on the south coast of Kenya, where a 75% reduction in the density of indoor resting *An. gambiae* mosquitoes was found in a setting where bed nets coverage reached high levels (60-86%) [38]. Similarly, the sporozoite index of *An. gambiae* recorded in Bozi and Yoho was much lower than the one reported in the control village. A comparable trend was found in a rural area of Tanzania, where the sporozoite index was lower in villages with high LLINs coverage, compared to villages without LLIN [50]. Furthermore, blood meals of mosquitoes were greatly disturbed by the presence of LLINs, as mosquitoes fed preferentially on animals and nectar plants [51].

The study suffers from two main limitations. First, seasonal trends could have influenced the values of the entomological indicators recorded; rainfall and temperature appear to be strongly correlated to vector abundance and EIR [52,53]. Second, in Côte d’Ivoire, resistance of *An. gambiae* to pyrethroids is widespread [54,55]; a study in Mozambique and South Africa reported failure in malaria control due to pyrethroid resistance [56]. Insecticides are used for net impregnation, and hence, this might have influenced the current findings. To limit the impact of both factors (season and resistance to insecticides), more villages should have been involved in the study. The entomological data reported here should be related to parasitological findings in school-aged children and this will be the subject of a subsequent publication.

To conclude, LLINs are an effective tool to fight malaria, as long as people use them properly. Despite the limitations stated above, the present study showed that by reinforcing households’ sensitization through continuous visits, increase LLINs usage is a real possibility to then reduce significantly *Plasmodium* transmission. This approach significantly impacted the EIR, including other key entomological indicators.

## Abbreviations

b/h/n: Bites per human per night; CHW: Community health worker; CI: Confidence interval; EIR: Entomological inoculation rate; ELISA: Enzyme-linked immunosorbent assay; f/h: Female per house; ib/h/n: Infectious bites per human per night; IRR: Incidence risk ratio; ITN: Insecticide-treated net; IRS: Indoor residual spraying; LLIN: Long-lasting insecticidal net; OD: Optical density; OR: Odds ratio.

## Competing interests

The authors declare that they have no competing interests.

## Authors’ contributions

AFO implemented the study, analysed and interpreted the data and drafted the manuscript. MD, GR and MT contributed to the design of the study and the revisions of the manuscript. EAVC and MK contributed to field activities. PLO assisted with the interpretation of the data and the revision of the manuscript. JU contributed to the design of the study and assisted in the interpretation of the data and the drafting and revision of the manuscript. BGK designed the study, coordinated field activities and assisted with data analysis and revision of the manuscript. All authors read and approved the final version of the manuscript prior to submission.

## Supplementary Material

Additional file 1 Abundance and specific composition of Culicidae fauna in N’Dakonankro. Click here for file

Additional file 2 Abundance and specific composition of Culicidae fauna in Yoho. Click here for file

Additional file 3 Abundance and specific composition of Culicidae fauna in Bozi. Click here for file
