# Supplementary material for: Transmission of malaria in relation to distribution and coverage of long-lasting insecticidal nets in central Côte d’Ivoire
Source: Malar J. 2014 Mar 19;13:109. doi: 10.1186/1475-2875-13-109 (PMC4000051; doi:10.1186/1475-2875-13-109)
Supplement: Additional file 1 — Abundance and specific composition of Culicidae fauna in N’Dakonankro. [file 1475-2875-13-109-S1.doc]

**Additional file 1.** Abundance and specific composition of Culicidae fauna in N’Dakonankro

|  | Date of survey | |  |  |  |  |
| --- | --- | --- | --- | --- | --- | --- |
| Species | July 2009 | July 2010 | August 2011 | November 2011 | February 2012 | Total |
| *Anopheles gambiae* | 276 (30.9) | 113 (27.4) | 383 (41.4) | 421 (79.6) | 972 (72.0) | 2,165 (56.9) |
| *Anopheles funestus* | 1 (0.1) | 9 (2.2) | 1 (0.2) | 0 | 0 | 11 (0.3) |
| *Anopheles nili* | 0 | 0 | 0 | 0 | 0 | 0 |
| *Anopheles pharoensis* | 61 (6.8) | 15 (3.6) | 42 (6.7) | 13 (2.5) | 94 (7.0) | 225 (5.9) |
| *Anopheles welcomei* | 305 (34.2) | 14 (3.4) | 18 (2.9) | 0 | 0 | 337 (8.8) |
| *Anopheles ziemani* | 3 | 1 (0.2) | 0 | 0 | 0 | 4 (0.1) |
| *Anopheles vittatus* | 0 | 0 | 0 | 0 | 1 (0.1) | 1 (0.0) |
| **Total *Anopheles*** | **646 (72.4)** | **152 (36.9)** | **444 (71.2)** | **434 (82.0)** | **1,067 (79.0)** | **2,743 (72.1)** |
| *Aedes aegypti* | 2 (0.2) | 1 (0.2) | 1 (0.2) | 0 | 2 (0.2) | 6 (0.2) |
| *Aedes palpalis* | 0 | 1 (0.2) | 3 (0.5) | 0 | 0 | 4 (0.1) |
| *Aedes africanus* | 0 | 0 | 0 | 0 | 0 | 0 |
| *Culex quinquefasciatus* | 54 (6.0) | 58 (14.1) | 59 (9.5) | 29 (5.5) | 261 (19.3) | 461 (12.1) |
| *Culex anulioris* | 6 (0.7) | 4 (1.0) | 3 (0.5) | 0 | 4 (0.3) | 17 (0.4) |
| *Culex cinerus* | 0 | 4 (1.0) | 0 | 0 | 0 | 4 (0.1) |
| *Culex decens* | 61 (6.8) | 0 | 0 | 1 (0.2) | 0 | 62 (1.6) |
| *Culex tigripes* | 0 | 0 | 0 | 0 | 0 | 0 (0) |
| *Mansonia africana* | 81 (9.1) | 59 (14.3) | 96 (15.4) | 33 (6.2) | 16 (1.2) | 285 (7.5) |
| *Mansonia uniformis* | 42 (4.7) | 133 (32.3) | 18 (2.9) | 32 (6.1) | 0 | 225 (5.9) |
| **Total other species** | **246 (27.6)** | **260 (63.1)** | **180 (28.8)** | **95 (17.9)** | **283 (21.0)** | **1,064 (27.9)** |
| **Total** | **892 (100)** | **412 (100)** | **624 (100)** | **529 (100)** | **1,350 (100)** | **3,807 (100)** |
