# Supplementary material for: Transmission of malaria in relation to distribution and coverage of long-lasting insecticidal nets in central Côte d’Ivoire
Source: Malar J. 2014 Mar 19;13:109. doi: 10.1186/1475-2875-13-109 (PMC4000051; doi:10.1186/1475-2875-13-109)
Supplement: Additional file 3 — Abundance and specific composition of Culicidae fauna in Bozi. [file 1475-2875-13-109-S3.doc]

**Additional file 3.** Abundance and specific composition of Culicidae fauna in Bozi

|  | Baseline situation before LLINs distribution | |  | Situation after LLINs  distribution | | |  |
| --- | --- | --- | --- | --- | --- | --- | --- |
| Species | July 2009 | July 2010 |  | August 2011 | November 2011 | February 2012 | Total |
| *Anopheles gambiae* | 85 (41.9) | 763 (88.5) |  | 3 (60.0) | 327 (95.1) | 17 (89.5) | 1,195 (83.4) |
| *Anopheles funestus* | 34 (16.7) | 4 (0.5) |  | 0 | 3 (0.9) | 0 | 41 (2.9) |
| *Anopheles nili* | 9 (4.4) | 0 |  | 0 | 1 (0.3) | 0 | 10 (0.7) |
| *Anopheles pharoensis* | 16 (7.9) | 66 (7.6) |  | 0 | 4 (1.2) | 0 | 86 (6.0) |
| *Anopheles welcomei* | 0 | 0 |  | 0 | 0 | 0 | 0 |
| *Anopheles ziemani* | 0 | 1 (0.1) |  | 0 | 0 | 0 | 1 (0.1) |
| *Anopheles vittatus* | 0 | 0 |  | 0 | 0 | 0 | 0 |
| **Total *Anopheles*** | **144 (70.9)** | **834 (96.8)** |  | **3 (60.0)** | **335 (97.4)** | **17 (89.5)** | **1,333 (93.0)** |
| *Aedes aegypti* | 0 | 4 (0.5) |  | 0 | 3 (0.9) | 0 | 7 (0.5) |
| *Aedes palpalis* | 0 | 1 (0.1) |  | 0 | 0 | 0 | 1 (0.1) |
| *Aedes africanus* | 0 | 0 |  | 0 | 0 | 0 | 0 |
| *Culex quinquefasciatus* | 16 (7.9) | 5 (0.6) |  | 1 (20.0) | 0 | 1 (5.7) | 23 (1.6) |
| *Culex anulioris* | 0 | 1 (0.1) |  | 0 | 0 | 1 (5.7) | 2 (0.1) |
| *Culex cinerus* | 0 | 0 |  | 0 | 0 | 0 | 0 |
| *Culex decens* | 5 (2.5) | 0 |  | 0 | 0 | 0 | 5 (0.3) |
| *Culex tigripes* | 0 | 0 |  | 0 | 0 | 0 | 0 |
| *Mansonia africana* | 28 (13.8) | 17 (2.0) |  | 1 (20.0) | 5 (1.5) | 0 | 51 (3.6) |
| *Mansonia uniformis* | 10 (4.9) | 0 |  | 0 | 1 (0.3) | 0 | 11 (0.8) |
| **Total other species** | **59 (29.1)** | **28 (3.2)** |  | **2 (40.0)** | **9 (2.6)** | **2 (10.5)** | **100 (7.0)** |
| **Total** | **203 (100)** | **862 (100)** |  | **5 (100)** | **344 (100)** | **19 (100)** | **1,433 (100)** |
